# A comparison of screw and suture button fixation in the management of adolescent ankle syndesmotic injuries

**DOI:** 10.1177/18632521241238889

**Published:** 2024-03-16

**Authors:** Luke Verlinsky, David M Heath, David A Momtaz, Boris Christopher, Aaron Singh, Steven D Gibbons

**Affiliations:** Department of Orthopaedics, UT Health San Antonio, San Antonio, TX, USA

**Keywords:** Syndesmosis, suture button, ankle fracture, syndesmotic screw

## Abstract

**Purpose:**

Ankle injuries involving the tibiofibular syndesmosis often necessitate operative fixation to restore stability to the ankle. Recent literature in the adult population has suggested that suture button fixation may be superior to screw fixation. There is little evidence as to which construct is preferable in the pediatric and adolescent population. This study investigates outcomes of suture button and screw fixation in adolescent ankle syndesmotic injuries.

**Methods:**

A retrospective matched cohort study over 10 years of pediatric patients who underwent ankle syndesmotic fixation at a large Level 1 Trauma Center was conducted. Both isolated syndesmotic injuries and ankle fractures with syndesmotic disruption were included. Preoperative variables collected include basic patient demographics, body mass index, and fracture type. Suture button and screw cohorts were matched based on age, race, sex, and open fracture utilizing propensity scores. Outcomes assessed include reoperation and implant failure.

**Results:**

A total of 44 cases of operative fixation of the ankle syndesmosis were identified with a mean age of 16 years. After matching cohorts based on age, sex, race, and open fracture status, there were 17 patients in the suture button and screw cohorts, respectively. Patients undergoing screw fixation had a six times greater risk of reoperation (p = 0.043) and 13 times greater risk of implant failure (p < 0.001). Out of six cases of reoperation in the screw cohort, five were unplanned.

**Conclusion:**

Our findings favor suture button fixation in operative management of adolescent tibiofibular syndesmotic injuries. Compared with screws, suture buttons are associated with lower risk of both reoperation and implant failure.

**Level of evidence:**

level III therapeutic.

## Introduction

The tibiofibular syndesmosis is a fibrous joint between the tibia and fibula that is integral to ankle stability.^1^ Injuries to the syndesmosis typically occur in the setting of excessive external rotation of the ankle coupled with dorsiflexion of the foot, resulting in pain and deformity. While most isolated injuries to the syndesmosis, colloquially called high ankle sprains, can be managed non-operatively, the most severe ligamentous injuries and those with concomitant fractures may require surgical reduction and fixation. Operative fixation of syndesmotic injuries traditionally was performed utilizing tricortical or quadricortical syndesmotic screws. However, the use of suture buttons as the primary fixation construct in the treatment of these injuries has gained significant popularity.

A recent randomized controlled trial by Andersen et al.^2^ found compelling evidence favoring suture button over screw fixation. In their study, the use of suture button fixation was associated with higher American Orthopaedic Foot & Ankle Society (AOFAS) scores and a higher likelihood of adequate reduction, defined in their study as a tibiofibular distance less than 2 mm between the injured and uninjured ankles, at both 1- and 2-year follow-up. Xu et al.^3^ found suture button fixation to be an equally effective technique and noted the distinct advantages with the technique, noting that suture buttons allow earlier weight-bearing and do not require routine removal. A systematic review and meta-analysis of 11 studies, 5 of which were randomized controlled trials, had similar conclusions and found a lower rate of postoperative complications with suture button fixation.^4^

Despite this, these studies are restricted to the adult population, limiting their generalizability to pediatric patients. As the number of pediatric patients participating in competitive sports increases, the need for pediatric focused literature does as well.^5^ Competitive sports are a common setting for ankle injuries, with high ankle sprains accounting for 7%–25% of these.^6^ Furthermore, ankles are the most common fracture location in young athletes and account for 5% of all fractures in the general pediatric population.^7,8^ Despite the risk and prevalence of syndesmotic injury in the pediatric patients, literature regarding operative management of these injuries is scarce.

The aim of this study is to compare rates of reoperation and mechanical implant failure in adolescent patients undergoing syndesmotic fixation with suture buttons or quadricortical syndesmotic screws. We hypothesize that the use of suture buttons will be associated with decreased incidence of reoperation and implant failure.

## Methods

### Study design and data source

This study is a retrospective analysis of electronic health record data collected from patients treated at a large academic practice associated with a Level 1 Trauma Center from 2012 to 2022. After institutional review board approval was obtained, the charts of 405 pediatric (less than age 18 years) patients who underwent operative fixation for ankle fractures were reviewed to identify patients who underwent surgical fixation of the tibiofibular syndesmosis with either suture button or syndesmotic screws. Syndesmotic injuries were either diagnosed preoperatively with plain radiographs demonstrating increased tibiofibular clear space widening or intraoperatively with the Cotton test and manual stress examination after fracture fixation. Preoperative variables were collected, including body mass index (BMI) and demographic information including patient sex, age, race, and ethnicity. Fracture type was also recorded. Postoperative clinical documentation was reviewed until the last clinical follow-up for the variables of interest.

### Surgical technique

**Figure 1.**
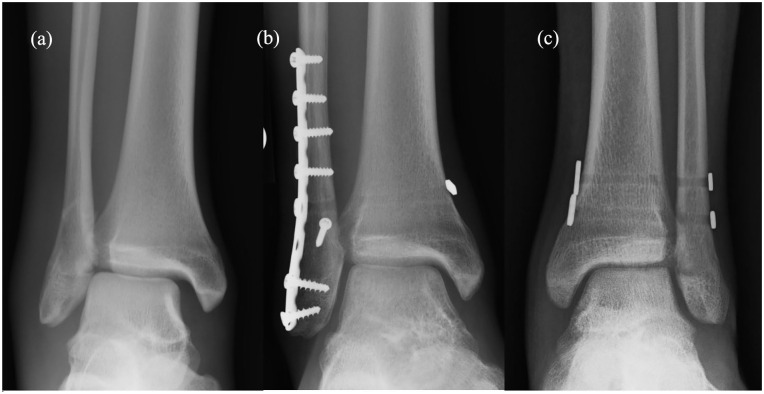
(a) Plain radiograph of Weber B type distal fibular fracture and associated syndesmotic disruption, (b) immediate postoperative radiograph of same patient treated with fibular fixation and suture button syndesmotic fixation, and (c) immediate postoperative radiograph of another patient who sustained isolated syndesmotic injury treated with two-suture button fixation constructs.

Patients with concomitant fractures and syndesmotic injuries underwent initial fracture fixation utilizing plate and screw constructs. After bony fixation was achieved, syndesmotic stress tests were performed utilizing manual stress and/or Cotton stress test. After syndesmotic injury was diagnosed intraoperatively, patients either underwent syndesmotic fixation with screw fixation or suture button fixation based on surgeon’s discretion. Syndesmotic reduction was performed either with syndesmotic reduction clamp or manual reduction. Either fixation device was placed perpendicular to the distal tibiofibular incisura to maintain anatomic reduction. For screw fixation, 3.5 mm quadricortical screws were utilized. For the suture button device, predrilling in a similar fashion was performed, and subsequently, the suture button was tensioned and deployed as dictated by the various implant specifications (Figure 1). Patients were allowed to begin range of motion exercises at 2 weeks and maintained non-weight-bearing status for a total of 6 weeks postoperatively.

### Statistical analysis

The total population was subsequently dichotomized with respect to treatment modality. The primary outcome of interest was reoperation. The incidence of mechanical implant failure was a secondary outcome of interest. Patients in the suture button and screw cohorts were matched based on age, sex, race, and open fracture using propensity scores with a nearest neighbor matching algorithm and a caliper of 0.1. Standard mean differences were assessed to verify covariate balance between cohorts after matching.

### Statistical models and measures of association and significance

Risk ratios compare the risk of an event in one group to the risk in another group. Absolute risks were initially for each cohort. Attributable risk analysis and risk ratio were offered where appropriate. Independent sample *t*-tests and the Wilcoxon rank-sum tests were performed for normally and non-normally distributed data, respectively. The Woolf–Haldane method was used to calculate risk ratios if one cohort offered zero contributions. Fisher’s exact test or chi-square with the Kendall tau was performed for categorical variables. Outcomes with a p-value < 0.05 were considered significant. All data were analyzed to ensure statistical integrity by a professional and double-checked by a senior team member.

### Statistical tools

International Business Machines (IBM) Statistical Package for the Social Sciences (SPSS) suite with Python package was used for data analysis. The University of California Los Angeles’ Advanced Research Computing Statistical Methods and Data Analysis G*Power Statistics tool was used for power analysis.

## Results

### Demographics

Of the 405 patient records reviewed, a total of 44 pediatric patients underwent syndesmotic fixation and thus met criteria for inclusion. Of these, 22 constructs utilized a syndesmotic screw and 22 utilized a suture button. After propensity score matching, 17 patients remained in each cohort. There was no difference in mean age between the screw cohort (16.5 years) and the suture button cohort (16.4 years). The mean BMI also did not differ between groups: 27 kg/m^2^ for the screw cohort and 24 kg/m^2^ for the suture button cohort. No significant differences in demographics were found between the two groups. Mean length of clinical follow-up was 335 days for the screw cohort and 209 days for the suture button cohort. While this difference did not approach significance (p = 0.091), there was a wide degree of variability in length of clinical follow-up. Demographic data can be seen in Table 1, while the AO Foundation/Orthopaedic Trauma Association (AO/OTA) fracture classifications and anatomic descriptions are depicted in Table 2.

**Table 1.** Patient demographics.

	Screw (N = 17)		Suture button (N = 17)		
	Mean	SD	Mean	SD	p
Age (years)	16.5	1.4	16.4	1.5	0.79
Follow-up (days)	334	218	209	202	0.091
BMI	27.7	8.5	24.4	13.0	0.39
	N	% (SE)	N	% (SE)	
Female	4	23.5% (10.3)	3	17.6% (9.2)	0.50
Black	1	5.9% (5.7)	3	17.6 (.92)	0.30
Hispanic	12	70.6% (11.1)	8	47.1 (12.1)	0.043

**Table 2.** Injury classifications for all study patients.

Injury characteristics	Screw (N = 22)	Suture button (N = 22)
	N (%)	N (%)
Isolated Fibula Fracture		
Weber B	6 (27)	4 (18)
Weber C	6 (27)	2 (9)
Isolated Medial Malleolus Fracture	0 (0)	2 (9)
Bimalleolar Fracture	5 (23)	6 (27)
Trimalleolar Fracture	2 (9)	2 (9)
Isolated Syndesmosis/Maisonneuve	2 (9)	5 (23)
Other	1 (1)	3 (14)

### Postoperative outcomes

**Figure 2.**
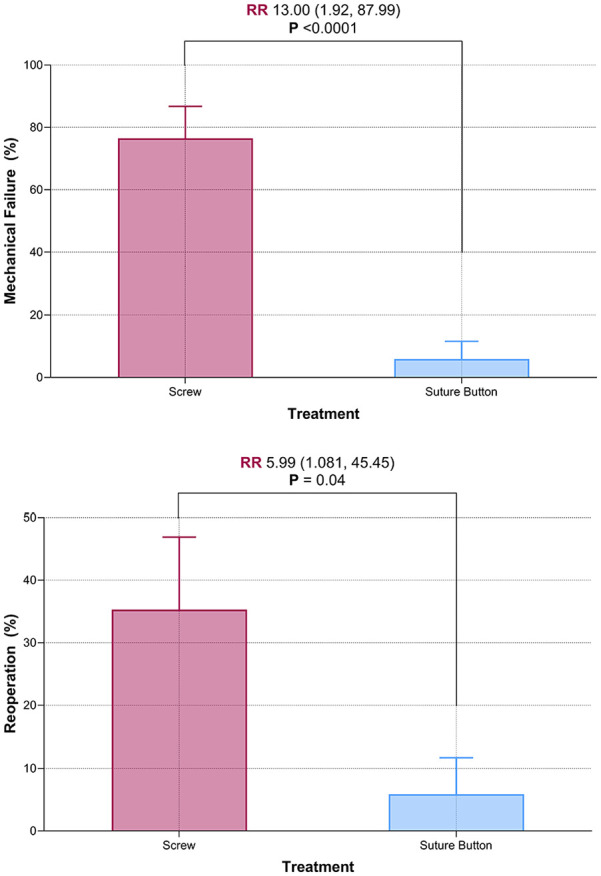
Box and whisker plots demonstrating risk ratios of mechanical implant failure and reoperation.

**Figure 3.**
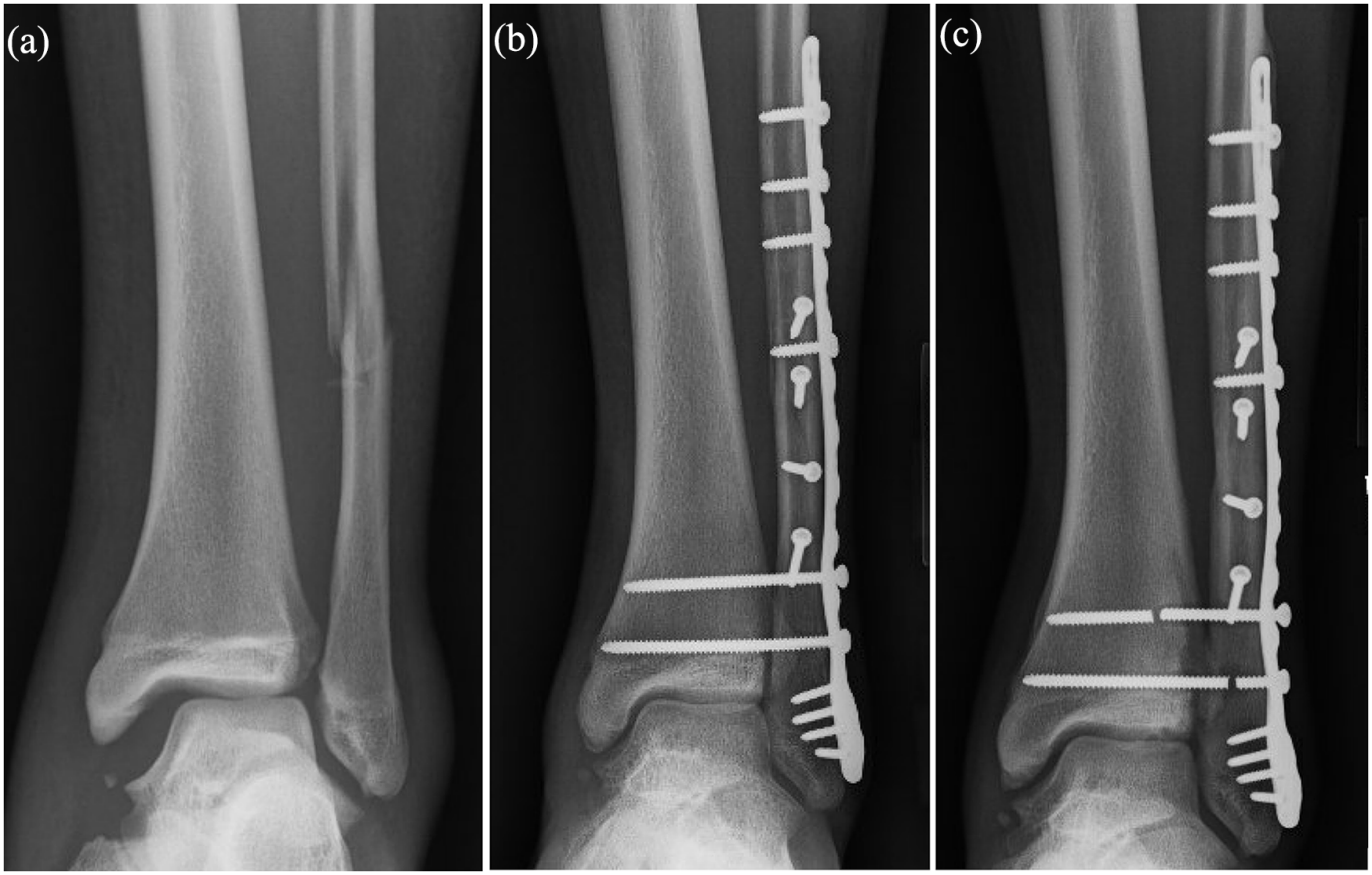
(a) Radiograph of Weber C type distal fibula fracture with medial clear space and tibiofibular clear space widening, (b) immediate postoperative radiograph of fibular plate and screw fixation construct and double syndesmotic screw fixation, and (c) 8-month postoperative radiograph demonstrating syndesmotic screw breakage requiring revision surgery.

We found that screw fixation was associated with greater risk of both reoperation and mechanical implant failure when compared with suture button fixation. A total of 6 (35%) patients required reoperation in the screw fixation cohort, compared with 1 (6%) in the suture button cohort (p = 0.043). A total of 13 patients with screws experienced mechanical implant failure (77%), while no suture buttons failed (p < 0.001) (Table 3). Screw fixation was associated with 13 times greater risk of mechanical implant failure and six times greater risk of reoperation (Table 4 and Figures 2 and 3). All mechanical failures of syndesmotic screws occurred after primary fracture union. None occurred prior to 6 weeks when patients were allowed to begin weight-bearing. Of note, the majority (5 of 6, 83%) of reoperations for syndesmotic screws were unplanned reoperation due to symptomatic implants as opposed to elective asymptomatic screw removal. Three of these patients reported deep ankle pain with weight-bearing that began after screw breakage, and two patients were revised for symptomatic prominent lateral plate fixation. Out of these five patients, four reported relief of symptoms postoperatively, and one was lost to follow-up. The only reoperation in the suture button group was due to symptomatic medial malleolus screws which were removed in isolation.

**Table 3.** Outcomes in matched cohorts.

	Screw (N = 17)		Suture button (N = 17)		
	N	% (SE)	N	% (SE)	p
Reoperation	6	35.3% (11.6)	1	5.9 (5.7)	0.043
Implant failure	13	76.5% (10.3)	0	0 (0)	<0.001

**Table 4.** Risk ratios and associated 95% confidence intervals.

	Relative risk	95% confidence interval
Mechanical Failure	13.00	(1.92, 87.99)
Reoperation	5.99	(1.08, 45.45)

## Discussion

Literature in the adult population on the use of suture button fixation of syndesmotic injuries is promising, as this construct allows earlier weight bearing, does not require routine removal, and results in equivalent or superior reduction when compared with syndesmotic screw constructs.^9^ Despite this, there is little that has been published on comparative outcomes in the pediatric population.^10,11^ This study aimed to assess outcomes associated with these two techniques in a pediatric population and found that screw fixation was associated with higher rates of reoperation and mechanical implant failure.

In a study assessing 77 adolescents (mean age 16 years), 32 undergoing suture button fixations to 45 undergoing screw fixations, Lurie et al.^10^ found that there was less need for reoperation, namely, implant removal surgery, in patients who underwent suture button fixation when compared with syndesmotic screw fixation (80% vs 12.5%, p < 0.001) but importantly did not distinguish between planned syndesmosis screw removal and removal for symptomatic implants or implant failure. Despite this, these findings corroborate our own and highlight a distinct advantage of suture button fixation. Reoperation places an additional burden on the patient, is associated with increased morbidity, and bears additional economic cost.^12–14^ In our study, we found that the majority (83%) of reoperations in the screw cohort were associated with symptomatic implants.

Lurie et al. also found no differences in functional scores at 4 years postoperative follow-up, suggesting that the two techniques were at least equivalent in this regard. Several studies in the adult population found superior outcomes with suture button fixation, suggesting additional benefit.^2,15,16^ Zhang et al.^15^ found that suture button fixation was associated with a better range of motion and allowed earlier return to work. After a 2-year follow-up period in a randomized trial, Andersen et al.^2^ found better AOFAS scores, fewer reports of pain, and less widening on radiographs in the suture button group. Similarly, after 5 years, Ræder et al.^16^ also found superior AOFAS scores and a lower incidence of ankle osteoarthritis. While these findings have not yet been reproduced in the pediatric population, they suggest that long-term outcomes may be improved.

The superior outcomes are likely due to improved reduction of the distal tibiofibular joint with the use of a flexible construct such as a suture button. Biomechanical studies suggest that suture button fixation may be associated with superior reduction of the distal tibiofibular joint, attributable to the dynamic stabilization achieved.^10,17^ In contrast, screw fixation provides more strength, but at the expense of a more rigid fixation that may result in malreduction.^17^ Furthermore, Jordan et al.^18^ found tibiofibular diastasis on radiographs after screw removal, suggesting that implant removal may impact the overall quality of the reduction, potentially impacting outcomes.

With reduced need for reoperation, suture button fixation may be a more cost-effective treatment option. Weber et al.^19^ found that routine removal of screws resulted in an additional $85,000 to $194,656 per 100 ankles in excessive cost. However, if screw removal was not necessary, suture buttons were less cost-effective on a patient-by-patient basis. Still, although many are moving away from routine screw removal,^20–22^ Neary et al.^23^ still found suture buttons to be more cost-effective. They suggest that screw fixation only becomes cost-effective when reoperation rates are below 10%. Ramsey and Friess^24^ concluded that suture button fixation was more cost-effective if reoperation rates for screw fixation exceeded 17.5% and screw fixation was more cost-effective until reoperation rates were below 13.7%. Both figures are below the on-demand removal rate of 23% reported by Sanders et al.^21^

With respect to mechanical implant failure rates, it is unclear if this outcome is of clinical significance. Screw breakage is common, particularly once the patient begins weight-bearing, and Hamid et al.^25^ found retained, broken screws are associated with superior outcomes relative to an intact or removed screw. However, more recent work by Ibrahim et al.^26^ suggested that screw breakage may be more serious than previously thought. Broken intraosseous screws were associated with increased odds of reoperation, typically for pain due to retained implants. Still, innovation in screw technology may soon address this issue. Stenquist et al.^27^ published preliminary results from a novel screw with a designed breakpoint aimed at minimizing pain and irritation from retained screws. The aim was to negate the need for routine removal and minimize the occurrence of on-demand removal for symptomatic implants. This may impact both cost and outcomes. Novel innovations in screw technology, such as this, necessitate further comparison between screw and suture button techniques. In our study, syndesmotic screws were found to have a higher rate of implant failure and a higher reoperation rate for deep ankle pain due to implant failure, which demonstrates clinical significance.

Our results favor the use of suture button fixation for syndesmotic fixation in adolescent patients, a demographic with a dearth of published outcomes. With reduced rates of mechanical implant failure, and more importantly, reoperation, suture buttons spare patients an additional operation and the associated costs and morbidity. In our cohort, patients who received syndesmotic screw fixation were more likely to undergo unplanned reoperation than those with suture buttons. The only patient in the suture button cohort who underwent reoperation had hardware removal of medial screws that were unrelated to the suture button construct. Still, with recent changes in practice patterns regarding routine screw removal and novel screw technology, further research is warranted to assess both cost and functional outcomes associated with both operative techniques.

### Limitations

Our study had several limitations. Our sample was drawn from a single institution and was relatively small. A significant number of patients were also lost to follow-up, potentially systematically excluding patients. Furthermore, the inclusion of patient reported outcome measures would make the study more compelling; however, the focus of this study was on reoperation rate and implant failure. Importantly, this study focused on the adolescent population. While our series had an average age of 16 years, which is on the older side for a pediatric study, this is intrinsic to this injury pattern. In our patient population, younger patients were more susceptible to the Salter–Harris fractures and transitional zone fractures such as Tillaux and Triplane fractures. These patients were excluded as they did not have syndesmotic disruption. As pediatric patients reach skeletal maturity, they are susceptible to these adult-type injury patterns but have been traditionally excluded from adult studies, which have inclusion criteria that start at 18 years of age. Future higher-powered, multi-center, and long-term studies are indicated in this unique patient population. Although we had follow-up greater than 1 year in each cohort, future prospective long-term outcomes studies would be particularly valuable as ankle fractures requiring syndesmotic fixation is associated with an increased risk for osteoarthritis.^28^

## Conclusion

Our findings favor the use of suture button fixation for tibiofibular syndesmosis injuries in adolescent patients. Suture button fixation was associated with lower risk of reoperation and mechanical implant failure when compared with traditional screw fixation. This spares the patient the burden, morbidity, and cost associated with an additional operation.

## Supplemental Material

sj-pdf-1-cho-10.1177_18632521241238889 – Supplemental material for A comparison of screw and suture button fixation in the management of adolescent ankle syndesmotic injuries Supplemental material, sj-pdf-1-cho-10.1177_18632521241238889 for A comparison of screw and suture button fixation in the management of adolescent ankle syndesmotic injuries by Luke Verlinsky, David M Heath, David A Momtaz, Boris Christopher, Aaron Singh and Steven D Gibbons in Journal of Children’s Orthopaedics
